# Mind the Gap: Gaps in Antidepressant Treatment, Treatment Adjustments, and Outcomes among Patients in Routine HIV Care in a Multisite U.S. Clinical Cohort

**DOI:** 10.1371/journal.pone.0166435

**Published:** 2017-01-26

**Authors:** Rushina Cholera, Brian W. Pence, Angela M. Bengtson, Heidi M. Crane, Katerina Christopoulos, Steven R. Cole, Rob Fredericksen, Bradley N. Gaynes, Amy Heine, W. Christopher Mathews, Matthew J. Mimiaga, Richard Moore, Sonia Napravnik, Conall O’Clerigh, Steven Safren, Michael J. Mugavero

**Affiliations:** 1 UNC School of Medicine, the University of North Carolina at Chapel Hill, Chapel Hill NC, United States of America; 2 Department of Epidemiology, Gillings School of Global Public Health, the University of North Carolina at Chapel Hill, Chapel Hill NC, United States of America; 3 Department of Medicine, School of Medicine, University of Washington, Seattle, WA, United States of America; 4 HIV/AIDS Division, San Francisco General Hospital, University of California, San Francisco, San Francisco, CA, United States of America; 5 Department of Psychiatry, School of Medicine, the University of North Carolina at Chapel Hill, Chapel Hill NC, United States of America; 6 Division of Infectious Diseases, Department of Medicine, School of Medicine, the University of North Carolina at Chapel Hill, Chapel Hill NC, United States of America; 7 UCSD, Department of Medicine, School of Medicine, University of California, San Diego, San Diego, CA, United States of America; 8 Harvard Medical School/Massachusetts General Hospital, Department of Psychiatry, Boston, MA, United States of America; 9 The Fenway Institute, Fenway Health, Boston, MA, United States of America; 10 Harvard School of Public Health, Department of Epidemiology, Boston, MA, United States of America; 11 Department of Medicine, School of Medicine, Johns Hopkins University, Baltimore, MD, United States of America; 12 Department of Psychology, University of Miami, Miami FL, United States of America; 13 Department of Medicine and UAB Center for AIDS Research, University of Alabama at Birmingham, Birmingham AL, United States of America; San Antonio Military Medical Center, UNITED STATES

## Abstract

**Background:**

Depression affects 20–30% of HIV-infected patients and is associated with worse HIV outcomes. Although effective depression treatment is available, depression is largely untreated or undertreated in this population.

**Methods:**

We quantified gaps in antidepressant treatment, treatment adjustments, and outcomes among US patients in routine HIV care in the nationally distributed CNICS observational clinical cohort. This cohort combines detailed clinical data with regular, self-reported depressive severity assessments (Patient Health Questionnaire-9, PHQ-9). We considered whether participants with likely depression received antidepressants, whether participants on antidepressants with persistently high depressive symptoms received timely dose adjustments, and whether participants achieved depression remission. We considered a cross-sectional analysis (6,219 participants in care in 2011–2012) and a prospective analysis (2,936 participants newly initiating CNICS care when PHQ-9 screening was active).

**Results:**

The cross-sectional sample was 87% male, 53% Caucasian, 25% African American, and 18% Hispanic; the prospective sample was similar. In both samples, 39–44% had likely depression, with 44–60% of those receiving antidepressants. Of participants receiving antidepressants, 20–26% experienced persistently high depressive symptoms; only a small minority of those received antidepressant dose adjustments. Overall, 35–40% of participants on antidepressants achieved full depression remission. Remission among participants with persistently high depressive symptoms was rare regardless of dose adjustments.

**Conclusions:**

In this large, diverse cohort of US patients engaged in routine HIV care, we observed large gaps in antidepressant treatment, timely dose adjustment to address persistently high depressive symptoms, and antidepressant treatment outcomes. These results highlight the importance of more effective pharmacologic depression treatment models for HIV-infected patients.

## Introduction

Depression is a highly prevalent comorbidity among people living with HIV and is associated with a range of negative clinical outcomes, including reduced antiretroviral treatment (ART) adherence and increased mortality.[1, 2] Effective depression treatment, potentially in combination with adherence supports, may be important to improve HIV treatment adherence and clinical outcomes.[3–5] Yet despite effective pharmacological and psychotherapeutic treatments,[6, 7] depression among HIV-infected individuals often goes unrecognized clinically[8] and, when recognized, is often not treated.[9, 10] Moreover, the depression treatment that is provided has rarely been characterized in detail.[11] In particular, to the authors’ knowledge no prior research among patients engaged in HIV care have distinguished *evidence-based* depression treatment from clinical inertia–the failure to initiate or intensify treatment when indicated on the basis of evidence-based guidelines.[12, 13] For pharmacological treatment, a core principle of modern evidence-based guidelines involves regular assessment of depressive symptom response, combined with promptly adjusting doses if depressive symptoms have not fully resolved.[14, 15]

Characterization of the gaps in depression identification, treatment, treatment adjustments, and response is central to guide development of mental health services for people living with HIV. Yet reliable estimates, especially from diverse populations in routine HIV care, are lacking. Here we estimate pharmacologic treatment gaps in a large, diverse multisite cohort of patients engaged in HIV primary care across the United States. We leverage a unique combination of detailed antidepressant medication histories and repeated, systematic depressive severity measures coinciding with HIV primary care appointments to characterize depression, antidepressant treatment, the extent to which persistently high depressive symptoms were followed by antidepressant adjustments, and antidepressant treatment outcomes. Guided by the conceptual framework described below,[14, 15] we hypothesized that the majority of patients with an indication for depression treatment would not be receiving pharmacologic treatment, the majority of treated patients with a need for antidepressant dose adjustment would not be receiving adjustments, and the majority of treated patients would not achieve timely depression remission.

## Methods

This study was approved by the Institutional Review Board at the University of North Carolina at Chapel Hill (IRB number 13–1707).

### Conceptual framework

Our approach was guided by the conceptual framework of the depression treatment continuum.[11, 16] Of patients engaged in HIV clinical care who also have depression, a portion are clinically recognized, a portion of those recognized initiate depression treatment, and a portion of those treated receive evidence-based treatment (primarily meaning structured psychotherapy of a given intensity and duration, or antidepressant treatment with careful monitoring and dose increases to achieve response[14, 15]), with sizeable drop-offs at each stage. While some depression will remit with only unstructured supportive counseling or low-dose antidepressant prescription, outcomes are expected to be better with evidence-based treatment, particularly given the primarily chronic nature of depression in the HIV-infected patient population.[17]

### Data source

We used the Center for AIDS Research (CFAR) Network of Integrated Clinical Systems (CNICS) observational cohort. The CNICS cohort includes over 30,000 HIV infected adults in care since 1995 at eight geographically diverse United States HIV clinical sites.[18] Approximately 1800 new patients enroll and 13% of existing patients leave the dynamic clinical cohort each year. Institutional review boards at each CNICS site have approved the study protocols.

The CNICS database captures comprehensive clinical data including standardized diagnosis, medication, laboratory, appointment, and demographic information collected through electronic health records and other institutional data systems. Medication data is based on a combination of electronic medical records, provider electronic order entry, and pharmacy databases. Prescription data is rigorously captured within each health system but each site also has protocols for capturing prescriptions from outside providers, although this data may not be as comprehensive as prescriptions issued by within-system providers. Data quality procedures have been described in more detail previously.[18] CNICS sites further collect self-administered socio-behavioral assessments, known as patient-reported outcomes (PROs). CNICS participants complete computer-based PROs at regular HIV primary care appointments, typically every 4–6 months.[19, 20] These questionnaires include validated measures of depression, anxiety, substance use, alcohol use, antiretroviral (ARV) adherence, sexual behavior, physical symptoms, and health-related quality of life. Although several of the CNICS sites do provide psychosocial services, data on these services is very limited. Data is not systematically collected on referrals to outside specialty mental health care.

### Measures

Depressive symptom severity was measured in the PROs by the Patient Health Questionnaire-9 (PHQ-9), a widely used screening tool that determines the presence and frequency during the previous two weeks of the 9 core symptoms of major depressive disorder.[21, 22] A PHQ-9 score ≥10 is generally considered a positive screen for likely major depression.[21, 22] The PHQ-9 is effective as a longitudinal measure of response to depression treatment, with a score <5 among those previously depressed or on depression treatment indicating remission (full treatment success).[23]

Antidepressant treatment was defined as a prescription for a selective serotonin reuptake inhibitor (citalopram, escitalopram, fluoxetine, fluvoxamine, paroxetine, sertraline), serotonin–norepinephrine reuptake inhibitor (desvenlafaxine, duloxetine, venlafaxine), or other second-generation antidepressant (bupropion, mirtazapine, nefazadone). Tricyclics alone (primarily amitriptyline in this sample) were not considered antidepressant treatment, as amitriptyline is often prescribed for pain relief in this population and tricyclics are uncommonly used currently as antidepressants. Tricyclic use was rare (<4% of person-time) in the analysis sample and half of tricyclic prescriptions were below minimally therapeutic doses for depression (e.g. ≤25mg daily of amitriptyline). Similarly, sleep agents alone (primarily trazodone in this sample) were not considered antidepressant treatment.

**Fig 1:**
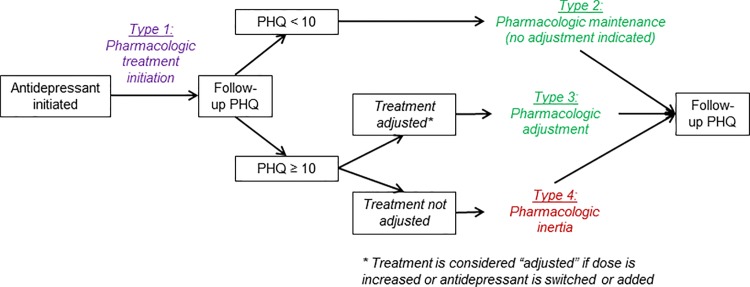
Conceptual framework for classification of antidepressant treatment decisions.

To characterize antidepressant treatment as *evidence-based* or not, we compared PHQ-9 scores and treatment decisions over time (Fig 1), capitalizing on the unique combination of longitudinal PHQ-9 measures and detailed medication histories. Evidence-based guidelines for antidepressant monitoring suggest that if significant depressive symptoms remain after ≥4–6 weeks on a given dose, the dose should be increased within the FDA-approved range if the medication is being tolerated.[14, 15] Therefore, if a PHQ-9 measure following antidepressant initiation was ≥10 and a treatment adjustment (dose increase or switch or addition of antidepressant) was subsequently made, we classified the period from the treatment adjustment to the next PHQ-9 measure as an episode of evidence-based antidepressant treatment (Fig 1, Type 3; pharmacologic adjustment). If no treatment adjustment was made, we classified the period as pharmacologic inertia, or the failure to make a change in antidepressant dose as suggested by evidence-based guidelines for pharmacologic depression treatment (Type 4If the PHQ-9 measure was <10 (meaning no treatment adjustment was indicated according to guidelines), the period until the next PHQ-9 was classified as evidence-based antidepressant treatment regardless of whether treatment adjustments occurred (Type 2; pharmacologic maintenance (no adjustment indicated)). The period from antidepressant initiation to the first follow-up PHQ-9 was classified separately as an initial treatment period (Type 1; pharmacologic treatment initiation).

### Statistical analysis

We considered two different perspectives: *cross-sectional*, representing the status of the active clinic patient population at a point in time; and *prospective*, representing the experience of a cohort of patients establishing care at CNICS sites.

#### Cross-sectional

Here, we restricted our attention to an 18-month period, July 2011-December 2012, during which time PRO collection was well established at 6 of 8 CNICS sites (Fenway Community Health Center-Harvard University; University of Alabama-Birmingham; University of California-San Diego; University of California-San Francisco; University of North Carolina-Chapel Hill; and University of Washington). For each PHQ-9 measure in this window we included the six following person-months in the analysis sample; one participant could have multiple PHQ-9s. For each person-month, we defined an *indication for depression treatment* as either a recent (within 6 months) PHQ-9 score ≥10 or a current antidepressant prescription. We defined *antidepressant treatment* as a current antidepressant prescription. We defined *evidence-based antidepressant depression treatment* as detailed above. We defined *remission of depression* as a PHQ-9 score <5 among those currently on antidepressant treatment.[23] We defined *mild* and *high depressive* symptoms as PHQ-9 scores of 5–9 and ≥10, respectively. We calculated the proportion of person-time meeting each definition to estimate period-prevalence of each characteristic.

#### Prospective

Here, we followed forward in time all participants newly establishing HIV care at a CNICS site after that site’s launch of PROs (ranging from 2005–2012). While all participants were new to care at CNICS sites, information was not always available on whether they were entirely new to HIV care or were transferring care. We defined *time to indication for depression treatment* as the time from entry to either (a) first PHQ-9 ≥10 or (b) antidepressant prescription. We defined *time to antidepressant treatment* as the time from entry to antidepressant prescription. We defined *time to remission* as the time from the first PHQ-9 ≥10 to the first subsequent PHQ-9 <5. Kaplan-Meier survival and failure functions for each of these endpoints were calculated, and the cumulative proportion “failing” (meeting criteria for depression, starting antidepressant treatment, achieving remission) was estimated at 12 months. Participants were censored after 12 months without an HIV medical appointment (lost to care) or at the site’s most recent CNICS data upload (administrative censoring). Using non-parametric Kaplan-Meier functions, we compared time to antidepressant treatment between person-time following a recent high PHQ-9 (≥10), following a recent low PHQ-9 (<10), or with no recent PHQ-9, with “recent” defined as within 6 months. In these comparisons, PHQ-9 status was treated as a time-varying characteristic: a participant’s person-time could be allocated to one or more of these categories over follow-up. Similarly, we compared time to remission between person-time receiving and not receiving antidepressant treatment.

To compare remission as a function of evidence-based antidepressant treatment vs. pharmacologic inertia, we divided each participant’s person-time treated with antidepressants into sequential “episodes” demarcated by each follow-up PHQ-9 measure, with each episode classified as one of Types 1–4 as defined above. We calculated the probability that episodes of each type ended with remission (PHQ-9<5). Episodes >1 year (i.e., no follow-up PHQ-9 measure within 1 year) were excluded. Once a participant had achieved remission, further episodes were excluded. Probabilities of remission and 95% confidence intervals were estimated using generalized estimating equations to account for correlation across multiple episodes per person.

In sensitivity analyses, we assessed the potential for bias due to non-random attrition in the prospective sample by applying inverse probability of observation weights based on variables available at the time of entry to care, including age, sex, race/ethnicity, HIV risk group, CD4 count, and viral load. As most sites do not administer PHQ-9s or other PROs at the first visit, these variables were not included in the weights. Results from weighted models were compared to results from the primary complete case analysis. Potential heterogeneity of estimates across sites was assessed with likelihood ratio tests comparing primary models to models that included a set of indicator terms for sites.

All analyses were conducted in Stata version 13 (College Station, Texas).

## Results

### Cross-sectional

#### Sample

Between July 2011-December 2012, 6,219 participants contributed 57,837 person-months of observation (mean [standard deviation]: 9.3 [5.1] per person) (Table 1). Most participants were male (87.2%) and the mean age was 45.3 (10.3) years. Over half of the participants were Caucasian (53%), a quarter were African-American (25%), and the majority of remaining participants were Hispanic (18%). Two-thirds were men who reported having sex with other men (MSM).

**Table 1:** Description of sample.

	Mean (SD) or n (%)	
	Cross-sectional	Prospective
Participants	6219 (100)	2936 (100)
Person-time per participant, mo.	9.3 (5.1)	29.6 (19.5)
Age, years	45.3 (10.3)	41.9 (10.9)
Current gender		
Male	5420 (87)	2575 (88)
Female	799 (13)	360 (12)
Transgendered	48 (1)	19 (1)
Race / ethnicity		
Caucasian non-Hispanic	3281 (53)	1465 (51)
African American non-Hispanic	1558 (25)	742 (26)
Hispanic	1097 (18)	538 (19)
Other	239 (4)	137 (5)
HIV transmission risk category		
Heterosexual sex	1161 (20)	381 (14)
MSM	3894 (65)	1843 (65)
IDU	819 (14)	538 (19)
Other	96 (2)	59 (2)
Site		
Site A	536 (9)	254 (9)
Site B	1655 (27)	753 (26)
Site C	101 (2)	3 (0)
Site D	2186 (35)	1135 (39)
Site E	819 (13)	89 (3)
Site F	922 (15)	702 (24)

#### Depression treatment continuum

**Fig 2:**
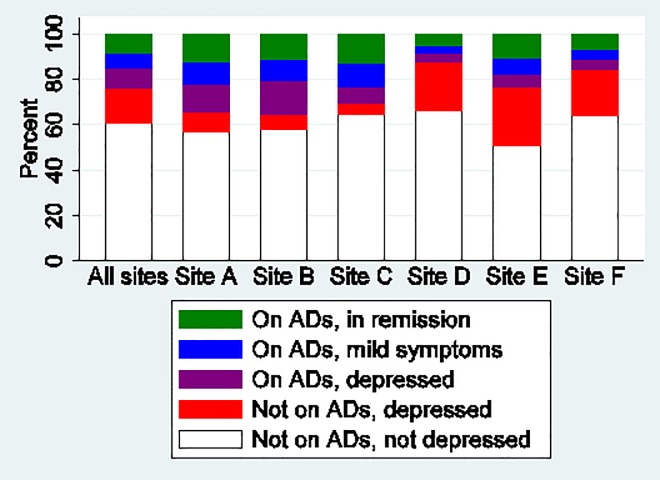
Distribution of depression and antidepressant treatment status across CNICS sites.

Across sites, 39% of person-time had an indication for depression treatment while 61% did not (Fig 2). For 16% of person-time, patients had a PHQ-9 score ≥10 and were not receiving antidepressants; for 9% of person-time, patients had a PHQ-9 score ≥10 and were receiving antidepressants; for 6% of person-time, patients had mild depressive symptoms (PHQ-9 score 5–9) while receiving antidepressants; and for 8% of person-time, patients were receiving antidepressants and were in remission (PHQ-9 score <5). Thus, of all patients with an indication for depression treatment, 60% were receiving antidepressant treatment, and of patients receiving treatment, 36% were in remission.

One-fifth of treated person-time (22%) was immediately following antidepressant initiation (Type 1) (Table 2). For half (52%) of treated person-time, no treatment adjustment was indicated because the patient was partially or fully responding to treatment (Type 2, pharmacologic maintenance (no adjustment indicated)). For the remaining one quarter (26%) of treated person-time, a treatment adjustment was indicated by ongoing high depressive symptoms; in about one-fifth of these cases (6% overall), a treatment adjustment was actually made (Type 3, pharmacologic adjustment), while in about four-fifths (20% overall) no treatment adjustment was made (Type 4, pharmacologic inertia).

**Table 2:** Antidepressant treatment, treatment adjustments, and remission.

	Distribution of person-time, %		Probability of remission, %
	Cross-sectional (4,831 PYO)	Prospective (7,240 PYO)	Prob (95% CI) (n/N)
No antidepressant treatment	76	79	n/a
Any antidepressant treatment	24	21	n/a
SSRI*	16	14	n/a
SNRI*	3	2	n/a
Other second-generation agent*	8	7	n/a
Multiple antidepressants*	4	3	n/a
Antidepressant treatment classification**			
Type 1, pharmacologic treatmentinitiation	22	58	23 (18–28) (52/230)
Type 2, pharmacologic maintenance	52	21	29 (18–40) (16/66)
Type 3, pharmacologic adjustment	6	5	0 (0–12) (0/28)
Type 4, pharmacologic inertia	20	15	9 (2–16) (4/68)

PYO: Person-years of observation. SSRI: Selective serotonin reuptake inhibitor. SNRI: Serotonin-norepinephrine reuptake inhibitor.

* Not mutually exclusive

** See Fig 1 legend

**Fig 3:**
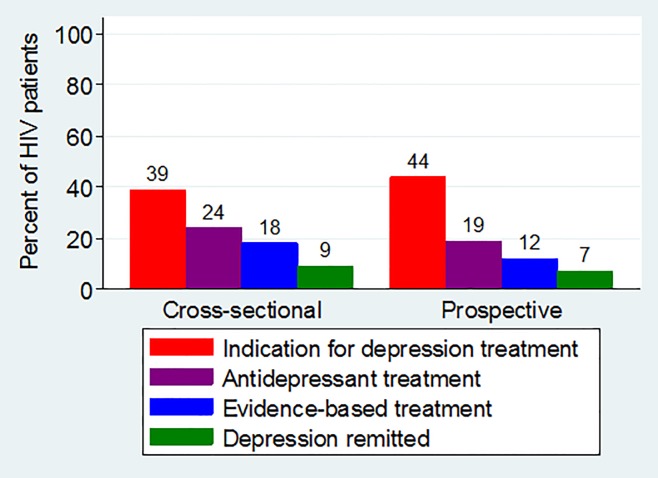
The depression treatment continuum for patients engaged in HIV primary care.

Combining these estimates indicates that 39% (95% CI: 39–40%) of the population had an indication for depression treatment, 24% (23–24%) were receiving antidepressant treatment, 18% (17–18%) were receiving evidence-based antidepressant treatment (either through passive pharmacologic maintenance or active pharmacologic adjustment), and 9% (9–10%) had achieved remission after starting pharmacologic treatment (Fig 3).

### Prospective approach

#### Sample

After the site-specific launch of PHQ-9 screening, 2,936 participants establishing HIV care contributed 86,906 person-months of observation (mean [SD}: 29.6 [19.5] per person) (Table 1). Demographic characteristics were similar to those of participants in the cross-sectional approach.

#### Depression treatment continuum

Among all participants new to CNICS care, the Kaplan-Meier failure function estimate of the proportion having an indication for depression treatment within the first 12 months of establishing HIV care at a CNICS site was 44%. The estimated probability of starting an antidepressant within the first 12 months of care was 44% in person-time following a PHQ-9 score ≥10, 17% in person-time following a PHQ-9 score <10, and 30% in person-time not immediately following a PHQ-9 screening. Among participants who had not started antidepressants at the time of PHQ-9 screening, the probability of achieving remission within 12 months of a PHQ-9 score ≥10 was 36% overall, 34% in person-time following antidepressant initiation, and 39% in person-time not following antidepressant initiation.

When considering evidence-based antidepressant treatment versus pharmacologic inertia, over half (58%) of the treated person-time was before the first follow-up PHQ-9 (Type 1; pharmacologic treatment initiation) (Table 2). Approximately one-fifth (21%) of person-time followed a PHQ-9 score <10, when no treatment adjustment would be indicated (Type 2, pharmacologic maintenance (no adjustment indicated). The remaining 20% of person-time followed a PHQ-9 score ≥10, when a treatment adjustment would be indicated; in approximately one-quarter of cases, or 5% overall, a pharmacologic adjustment was made (Type 3,pharmacologic adjustment) whereas in three-quarters of cases, or 15% overall, no treatment adjustment was made (Type 4, pharmacologic inertia). The probability of remission at the end of the initial treatment period (Type 1) was 23% (95% CI: 18–28%) and at the end of pharmacologic maintenance episodes where no adjustment was indicated (Type 2) was 29% (95% CI: 18–40%). At the end of the 28 episodes of evidence based antidepressant treatment where pharmacologic adjustment was made (Type 3), there were no instances of remission (0%; exact 95% CI: 0–12%) and at the end of the 68 episodes of pharmacologic inertia (Type 4), the probability of remission was 9% (2–16%).

Combining these estimates indicates that 44% (42–46%) of the population had an indication for depression treatment in the first 12 months of CNICS care, 19% (17–22%) received any antidepressant treatment in the first 12 months, 12% (11–14%) were receiving evidence-based antidepressant treatment (either through passive pharmacologic maintenance or active pharmacologic adjustment), and 7% (6–8%) achieved remission within 12 months (Fig 3). There was notable variation in the cascade parameters across sites: the proportion having an indication for depression treatment ranged from 37–46%, the proportion receiving any antidepressant ranged from 14–30%, the proportion receiving evidence-based treatment ranged from 6–20%, and the proportion achieving remission ranged from 2–9%.

In sensitivity analyses to assess the role of non-random attrition, estimates of the depression treatment continuum (Fig 3) and the probability of remission (Table 2) were virtually unchanged when inverse probability of observation weights were applied.

## Discussion

This analysis represents the first comprehensive assessment of the depression treatment continuum in a large, diverse sample of US patients engaged in HIV primary care. We identified substantial gaps in pharmacologic depression treatment, antidepressant adjustments, and remission in this population. Regardless of whether we considered a cross-section of all active clinic patients or a prospective cohort of patients establishing care, approximately 2 in 5 patients were in need of depression treatment, about half of those in need received antidepressant treatment, and fewer than half of those on antidepressant treatment achieved full depression remission.

Approximately 40% of CNICS patients had an indication for depression treatment. This estimate is consistent with other reports of depression prevalence among HIV-infected patients in similar primary care populations.[10, 24–32] Of the patients in our analyses who had an indication for depression treatment, the probability of receiving antidepressants was 43% among patients newly establishing care, and 60% in our cross-sectional analysis. These estimates are generally comparable to those from similar settings, in which the prevalence of antidepressant treatment among HIV-infected patients has ranged from 40–49%.[9, 10, 33, 34]

The combination in the CNICS cohort of repeated depressive severity assessments at clinical contacts paired with detailed antidepressant medication histories provided a unique opportunity to evaluate antidepressant treatment adjustments in this population. We assessed the frequency with which patients on antidepressant treatment but with persistently high depressive symptoms received dose adjustments as indicated by evidence-based guidelines.[12, 13] While we estimated that more than half of treated person-time was evidence-based antidepressant treatment, in the vast majority of cases this was because the patient had no or mild depressive symptoms and no treatment adjustment was indicated Of the patients who required active evidence-based antidepressant treatment (Types 3 and 4) because of persistent high depressive symptoms, only one in four received a pharmacologic treatment adjustment. Importantly, the database does not identify the prescriber; some antidepressants may have been prescribed by a psychiatrist rather than the HIV provider. Nevertheless, this result suggests that prescribers for these patients may be comfortable with initiating antidepressant medications but may not titrate when the depressive illness fails to respond to an initial dose. Care models that support primary care physicians in prescribing antidepressants may be necessary to address this gap.[35–37]

Among the small number of patients in our analysis who required and received antidepressant treatment adjustment, none achieved remission; among patients who required treatment adjustment and did not receive it, remission rates were not much different. In contrast, about a quarter of patients achieved remission within the initial antidepressant treatment period, and of patients exhibiting mild remaining depressive symptoms while on treatment, about a quarter went on to achieve remission. In addition, after a high PHQ-9, the overall remission rates between patients starting and not starting antidepressant treatment were quite similar. These findings suggest two separate depression phenotypes- one that responds promptly to antidepressant treatment, and may even resolve in the absence of treatment (for example, newly diagnosed patients in whom high depressive symptoms may reflect short-term adjustment disorder rather than a major depressive episode), and one that is treatment-resistant regardless of antidepressant treatment adjustments. These phenotypes would almost certainly be associated with a range of covariates including medical and psychiatric comorbidities and psychosocial factors. It is possible for example that the patients who received treatment adjustments were those with psychiatric complexity or otherwise known to be treatment-resistant and were referred to psychiatric resources. These patients would have been less likely to achieve remission due to their depression phenotype, a type of confounding by indication, perhaps explaining why none of the patients who received Type 3 treatment achieved remission.

The limitations of this study should be considered. First, depression and remission are defined based on a screening instrument that, while possessing excellent psychometric properties, is not diagnostic. To benchmark the performance of the PHQ-9 screening tool in this study relative to other populations, our results can be compared to data from the STAR*D randomized controlled trial in which the QIDS-SR16, a screening tool with similar properties to the PHQ-9, was used to assess depression in a large non-HIV infected outpatient sample.[38] In that study the depression remission rate was 36% after initial treatment initiation, compared to 23% in the analogous time period in the current study. This comparison of two large populations in which depression was measured with similar self-report screening tools suggests that there may be a lower remission rate in this HIV-infected cohort compared to non-HIV infected outpatient populations. Second, the measure of pharmacologic depression treatment used in these analyses was imperfect for several reasons. We made the assumption that patients obtained and were adherent with antidepressant medications as they were prescribed. As prescription uptake and adherence would be less than 100%, treatment was likely overestimated, although providers’ clinical decisions were accurately captured. Third, these data do not provide information about the reasons why some patients with persistent high depressive symptoms did not receive a dose adjustment. For example, a dose adjustment may have been suggested by the provider but declined by the patient. The provider may not have been aware of the PHQ-9 result (although the PHQ-9 score as a measure of the patient’s depressive severity on that day is nonetheless valid). Similarly, the provider may have based a dosing decision on other factors gathered during the clinical encounter not captured by the PHQ-9. Importantly, we focused specifically on pharmacological treatment of depression; other important and effective depression treatments, particularly evidence-based psychotherapy, were not included in this analysis. In this regard treatment may have been underestimated if a large proportion of the study population engaged in psychotherapeutic treatment. While many Type 4 episodes in this analysis may be non-evidence based treatment periods, in that patients needing a pharmacologic treatment adjustment did not receive a dose adjustment, we cannot draw definitive conclusions about the proportion of these episodes that represent true gaps in depression treatment without information on other modalities of depression treatment such as psychotherapy. Fourth, estimates may be biased due to non-random attrition. While analyses accounting for possible differential attrition by demographic and clinical variables were nearly identical to primary analyses, we did not have measures of depressive severity at entry to care for most patients. If patients with depression at entry were most likely to be lost to care before completing a PHQ-9, our estimates of depression prevalence would be biased downward. Finally, while CNICS contains comprehensive medication information from within each site’s health system, and all sites have clinical protocols that include capturing prescriptions from outside providers during clinic appointments, prescriptions by outside providers may still be under-represented in the database.

Another important consideration is the irregular timing and spacing of depressive severity and remission measures used in our analyses. In a real-world outpatient psychiatric setting, depressive severity is ideally measured within 4–6 weeks of initiating antidepressant treatment and appropriate changes to the treatment regimen are made. In this study we did not have such frequent measures of depression so we considered severity and remission assessments up to 6 months after antidepressant initiation. While our results provide important aggregate parameters of the pharmacologic depression treatment continuum within the HIV clinical setting, estimates of remission may not reflect the detailed course of illness assessed in psychiatric care settings. However, our findings may be highly germane to the provision of pharmacologic depression treatment in outpatient HIV care settings.

A key strength of this study is the size, diversity, and richness of the data source. The CNICS cohort is distinct from many other longitudinal HIV cohorts in that it is a clinical care cohort: participation requires only consent to have routinely collected medical data captured, leading to very high participation rates across all sites. With inclusion of medical centers in the West, South, mid-Atlantic, and Northeast, the relevance of the experiences of the CNICS cohort to patients engaged in HIV primary care across the US is high. At the same time CNICS is distinct from many administrative databases in its inclusion of a range of validated self-reported behavioral assessments that have been integrated into routine clinical care, permitting investigations such as the current one that require measures of depressive severity linked in time to clinical actions. It is important to note that CNICS sites have established rapid clinical assessment and response protocols for patients indicating suicidality on the PROs, which may have led to better depression identification and treatment in the CNICS cohort than would have been present without the PROs. One interesting observation from the present analysis was the range in depression treatment and outcome indicators across sites. Although it was beyond the scope of this analysis to explore reasons for this heterogeneity, such differences could be due to differences in care practices or differences in patient populations (e.g. insurance coverage or cultural attitudes towards antidepressant prescription).

This study suggests substantial gaps across the steps of the depression treatment continuum among patients in HIV primary care settings across the United States and provides the first estimates of evidence-based antidepressant treatment delivery in these settings. Our findings show that successful pharmacologic treatment of depression is uncommon among patients with HIV infection, with small proportions of depressed patients receiving antidepressant dose adjustments when indicated or achieving remission. With increasing emphasis on decentralizing depression care from specialty mental health settings into primary care, including HIV primary care, HIV providers may need improved support in recognizing and treating depression, particularly in cases that require titrating or changing antidepressant treatment regimens. Collaborative care models that address these gaps in pharmacologic depression treatment could play an important role in addressing the burden of depression among patients with HIV infection.
